# Supplementary material for: Motility and tumor infiltration are key aspects of invariant natural killer T cell anti-tumor function
Source: Nat Commun. 2024 Feb 9;15:1213. doi: 10.1038/s41467-024-45208-z (PMC10853287; doi:10.1038/s41467-024-45208-z)
Supplement: Supplementary file 1 — Supplementary Information [file 41467_2024_45208_MOESM1_ESM.pdf]

Supplementary Information

Motility and tumour infiltration are key aspects of invariant natural killer T cell anti-tumour function

Chenxi Tian[1], Yu Wang[1], Miya Su[1], Yuanyuan Huang[1], Yuwei Zhang[1], Jiaxiang Dou[2], Changfeng Zhao[1], Yuting Cai[1], Jun Pan[1], Shiyu Bai[1], Qielan Wu[1], Sanwei Chen[3], Shuhang Li[1], Di Xie[1], Rong Lv[4], Yusheng Chen[2], Yucai Wang[1,5], Sicheng Fu[1,*], Huimin Zhang[1,*], Li Bai[1,2,5,6,*]

[1]Hefei national Research Center for Physical Sciences at the Microscale, Center for Advanced Interdisciplinary Science and Biomedicine of IHM, Division of Life Sciences and Medicine, University of Science and Technology of China, Hefei, China.

[2]Institute of Health and Medicine, Hefei Comprehensive National Science Center, Hefei, China.

[3]The First Affiliated Hospital of Anhui Medical University, Hefei, China.

[4]Anhui Blood Center, Heifei, China.

[5]Biomedical Sciences and Health Laboratory of Anhui Province, Division of Life Sciences and Medicine, University of Science and Technology of China, Hefei, China.

[6]National Synchrotron Radiation Laboratory, University of Science and Technology of China, Hefei, China.

[*]Correspondence to Sicheng Fu (fsc@mail.ustc.edu.cn), Huimin Zhang (hmzhang@ustc.edu.cn), and Li Bai (baili@ustc.edu.cn; ORCID: 0000000290281920).

The PDF file includes:

Supplementary Fig. 1 to 8

Supplementary Table

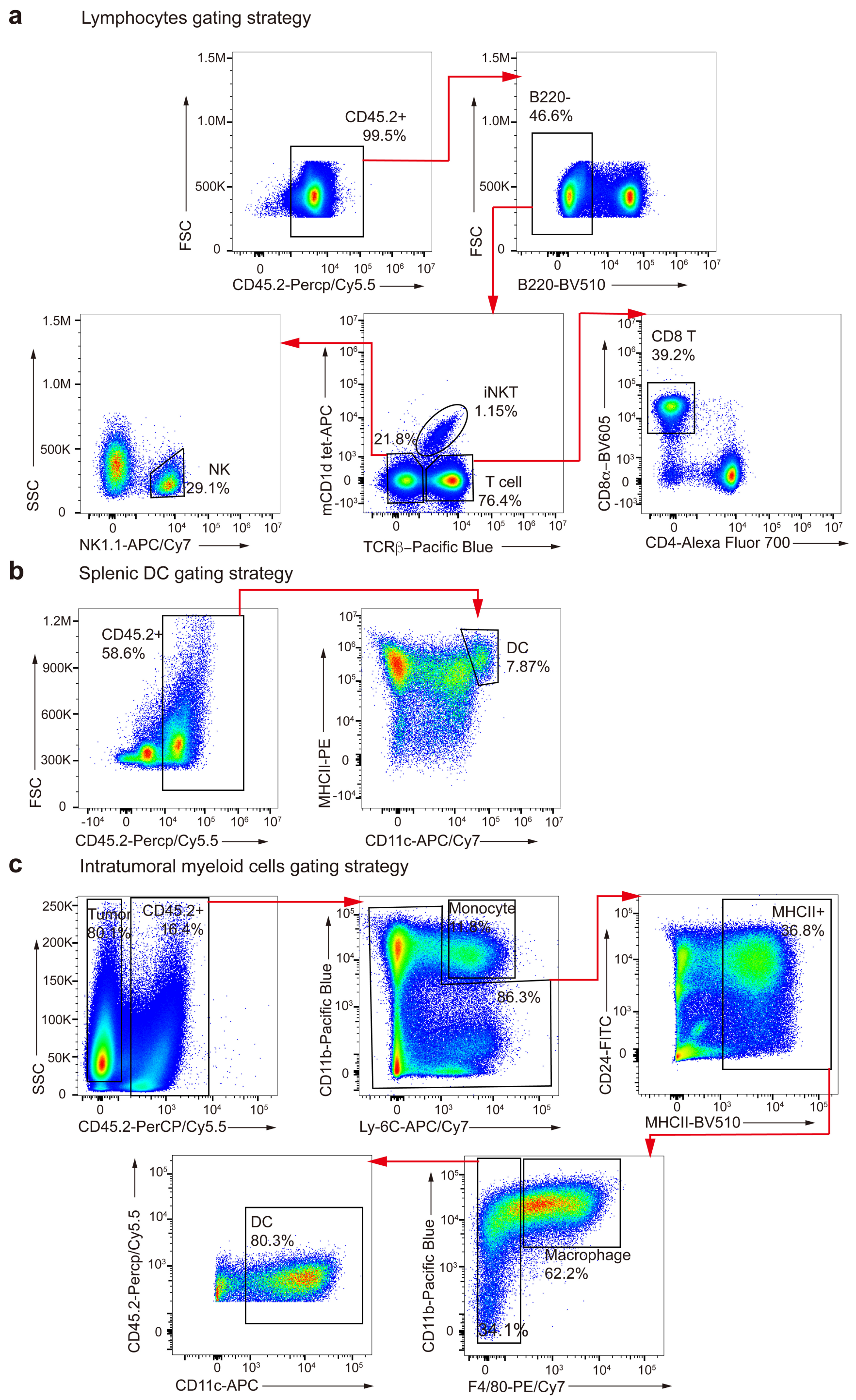

Supplementary Fig. 1 Gating strategy for lymphocytes and myeloid cells. a, Gating strategy for identification of indicated lymphocytes including NK cells, iNKT cells, and CD8 T cells. **b**, Gating strategy for identification of splenic DCs. **c,** Gating strategy for identification of intratumoral myeloid cells including monocytes, DCs, and macrophages. The gating strategy were used in Fig. 2, Fig. 3, Fig. 4, Fig. 5, Fig. 6, Fig. 7, Fig. 8, Supplementary Fig. 5, Supplementary Fig. 6, Supplementary Fig. 7.

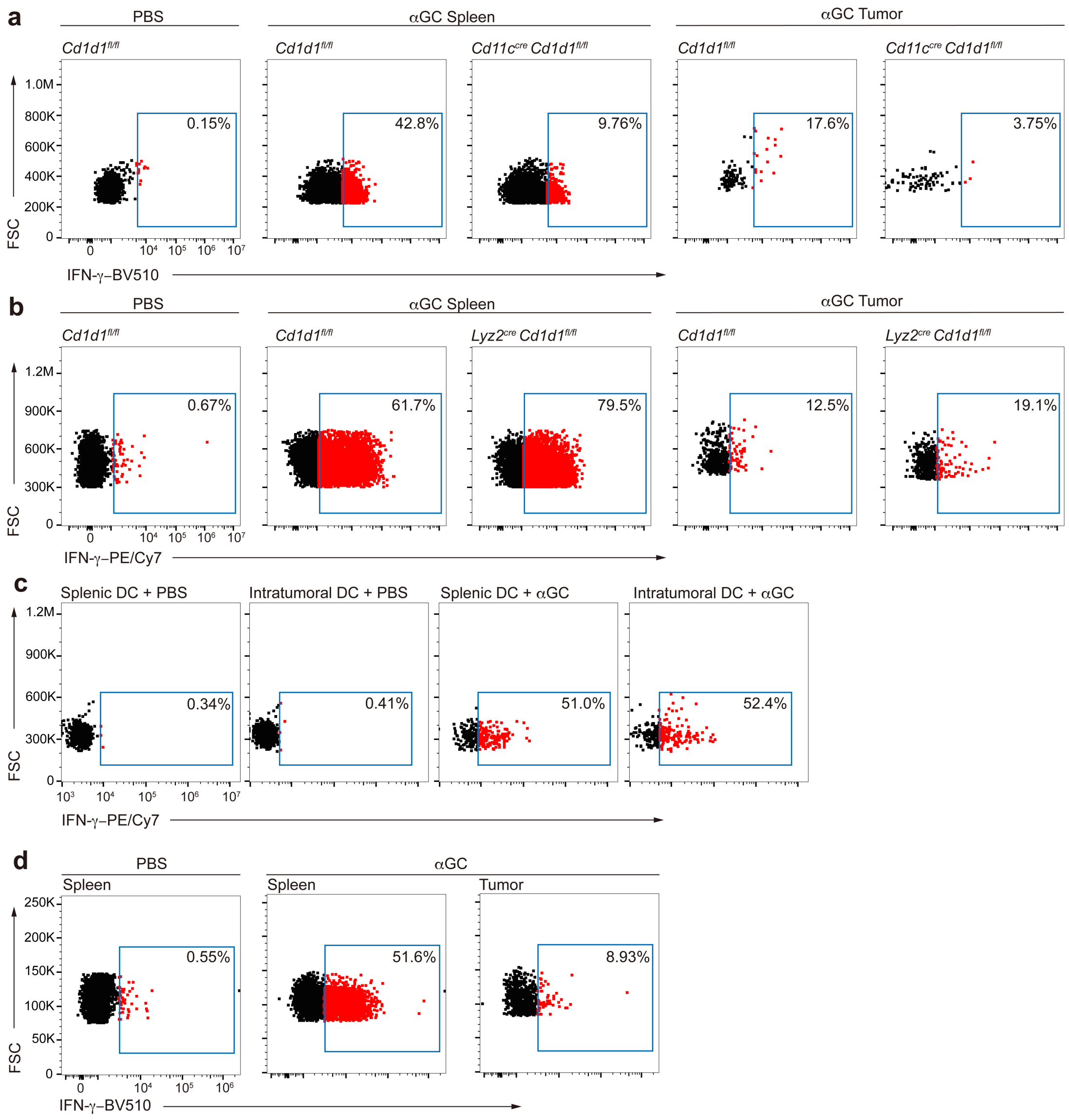

Supplementary Fig. 2 Intratumoral DCs fail to activate iNKT cells efficiently *in vivo* despite their normal antigen presentation capacity. a, Flow cytometry dot plots of IFN-γ production in splenic iNKT cells and intratumoral iNKT cells from MC38 tumor-bearing *Cd11c*^{cre} *Cd1d1*$^{fl/fl}$ mice and *Cd1d1*$^{fl/fl}$ mice, after αGC injection. **b**, Flow cytometry dot plots of IFN-γ production in splenic iNKT cells and intratumoral iNKT cells from MC38 tumor-bearing *Lyz2*^{cre} *Cd1d1*$^{fl/fl}$ mice and *Cd1d1*$^{fl/fl}$ mice, after αGC injection. **c**, Flow cytometry dot plots of IFN-γ production in hepatic iNKT cells activated by splenic or intratumoral DCs pre-loaded with PBS or αGC for 12 hours *in vitro*. **d**, Flow cytometry dot plots of IFN-γ production in splenic iNKT cells and intratumoral iNKT cells from MC38 tumor-bearing WT mice, after αGC injection. Supplementary Fig. 2 corresponds to Fig. 2b, Fig. 2c, Fig. 2d, Fig. 2f.

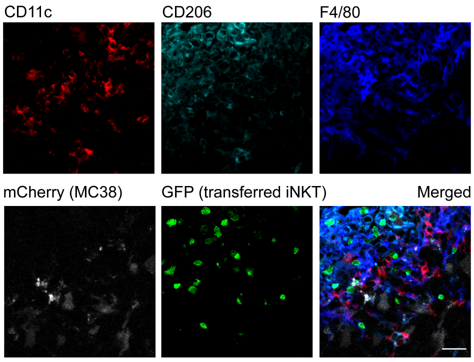

Supplementary Fig. 3 Characterization of macrophages in tumors. Staining of CD11c, CD206, F4/80 in MC38-mCherry tumors transferred with GFP^{+} iNKT cells (*n* = 4). Scale Bar, 20 μm. Data are representative of four independent experiments.

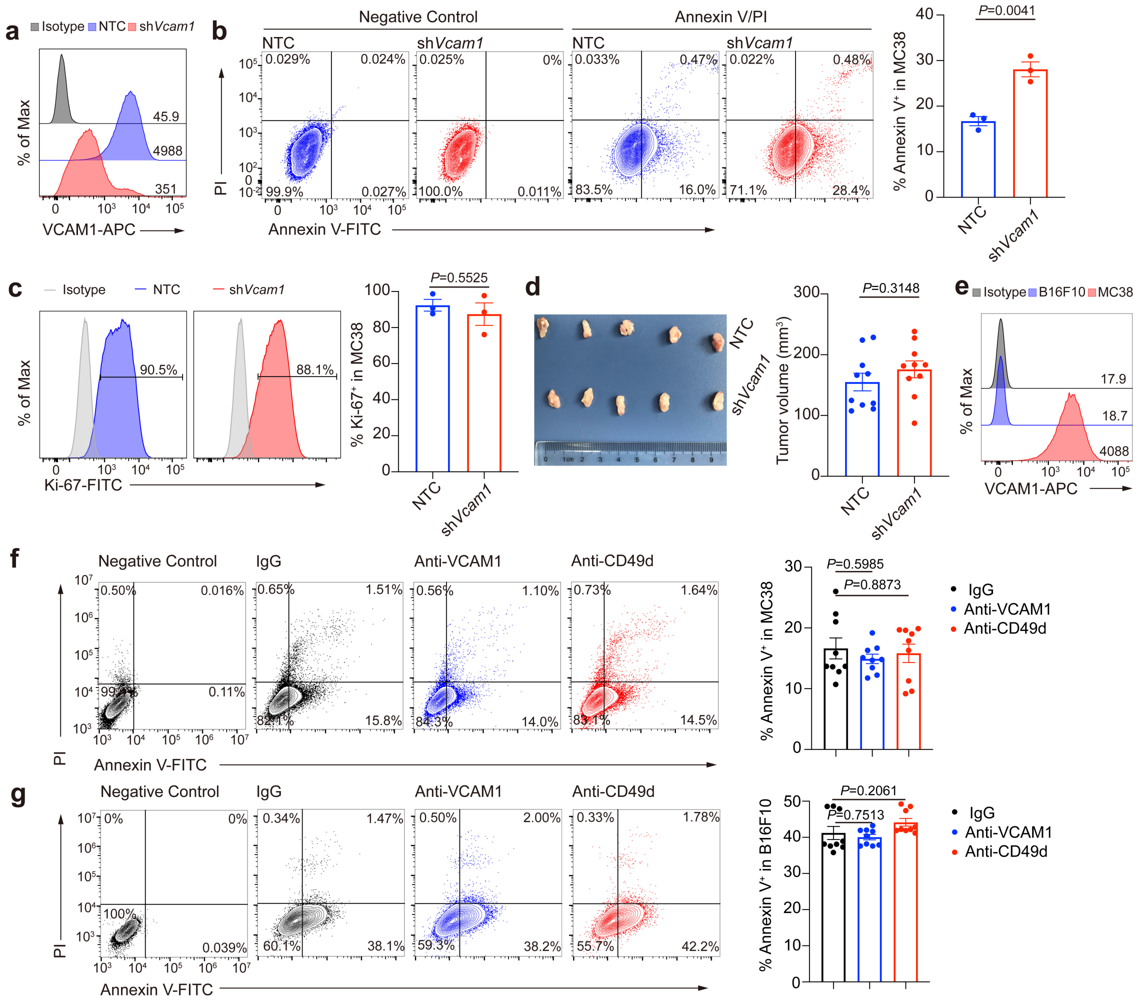

Supplementary Fig. 4 Different influences of *Vcam1* knockdown and antibody blockage on tumor cell apoptosis. a, VCAM1 expression in *Vcam1* knockdown MC38 cells and NTC MC38 cells. NTC, none target control. **b-c**, Influences of *Vcam1* knockdown on apoptosis (**b**) and proliferation (**c**) of MC38 cells (n = 3). **d**, Picture and weight of *Vcam1* knockdown MC38 tumors and NTC MC38 tumors, after injecting 1.5 × 10^6 *Vcam1* knockdown MC38 cells or 1 × 10^6 NTC MC38 cells into WT mice for 8 days (n = 10 mice per group). **e,** VCAM1 expression in B16F10 and MC38 tumor cells. **f-g**, Apoptosis of MC38 (**f,** n **=** 9) and B16F10 (**g,** n = 9) tumor cells treated with IgG isotype control antibody, anti-VCAM1 antibody or anti-CD49d antibody. Data are represented as mean ± SEM. Data are representative of (**a-g**) or pooled from (**b-d, f-g**) three independent experiments. Data were analyzed by two-tailed unpaired Student's t-test (**b-d**) and one-way ANOVA (**f, g**). Source data are provided as a Source Data file.

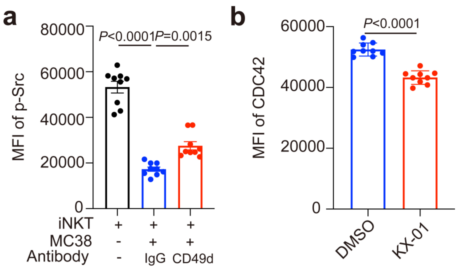

Supplementary Fig. 5 VCAM1-CD49d signaling reduces CDC42 expression in iNKT cells through Src signaling pathway. a, Phosphorylation of Src in iNKT cells co-cultured with or without MC38 tumor cells in the presence of anti-CD49d antibody or IgG isotype control antibody for 24 hours (n = 9). **b**, CDC42 expression in iNKT cells in the presence of DMSO or Src inhibitor KX-01 (2 μM) for 24 hours (n = 9). Data are pooled from three independent experiments (**a-b**). Data are represented as mean ± SEM, and were analyzed by one-way ANOVA (**a**) and two-tailed unpaired Student's t-test (**b**). Source data are provided as a Source Data file.

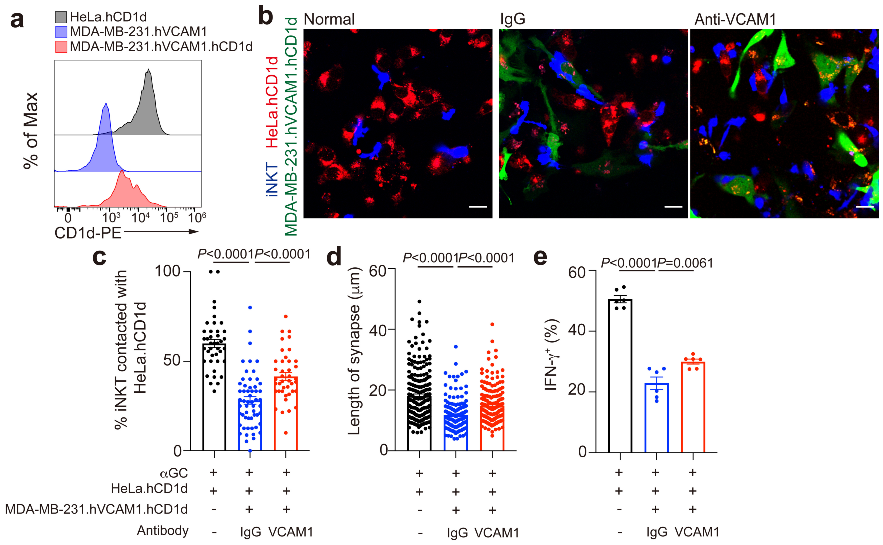

Supplementary Fig. 6 VCAM1 in CD1d-expressing tumor cells inhibits human iNKT cell activation. a, Expression of CD1d in Hela.hCD1d, MDA-MB-231.hVCAM1, and MDA-MB-231.hVCAM1.hCD1d cells. **b-d,** Images (**b**), frequencies (**c**, $n = 40, 56, 41$), and lengths of synapses (**d**, $n = 225, 183, 196$ cells) of CTV-stained expanded human iNKT cells contacted with CMTPX-stained αGC-pulsed HeLa.hCD1d cells, in the absence or presence of MDA-MB-231.hVCAM1.hCD1d cells and indicated antibodies. Scale Bars, 20 μm. **e**, IFN-γ production in expanded human iNKT cells activated as in **b-d** ($n = 6$). Data are representative of (**a-b**) or pooled from two independent experiments (**b-e**). Data are represented as mean ± SEM, and were analyzed by one-way ANOVA (**c-e**). Source data are provided as a Source Data file.

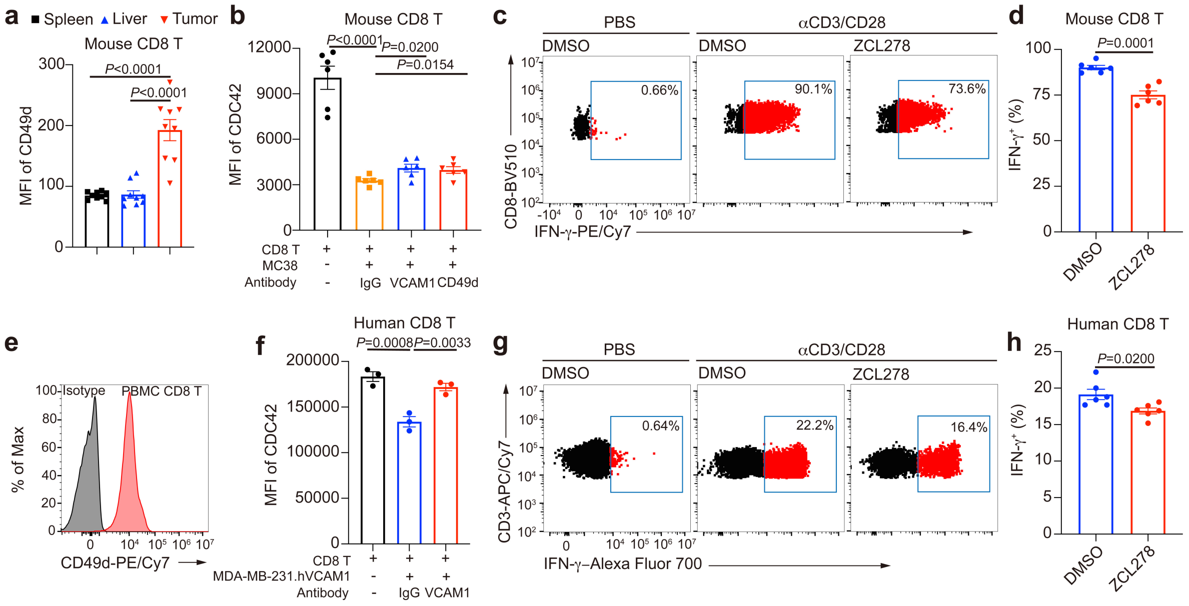

Supplementary Fig. 7 Interfering with VCAM1 signaling promotes CDC42 expression and activation of mouse and human CD8 T cells. a, CD49d expression in CD8 T cells from MC38 tumor-bearing WT mice (*n* = 9 mice). **b,** CDC42 expression in mouse CD8 T cells co-cultured with or without MC38 cells in the presence of anti-VCAM1 antibody, anti-CD49d antibody, or IgG isotype control antibody for 24 hours (*n* = 6 mice). **c-d**, Flow cytometry dot plots (**c**) and frequencies (**d**, *n* = 6 mice) of IFN-γ production in mouse CD8 T cells stimulated by plate-coated anti-CD3 (10 μg ml^{-1}) and anti-CD28 (10 μg ml^{-1}) in the presence of DMSO or ZCL278. **e**, CD49d expression in human CD8 T cells from PBMC. **f,** CDC42 expression in human CD8 T cells co-cultured with or without MDA-MB-231.hVCAM1 cells in the presence of anti-VCAM1 antibody or IgG isotype control antibody for 24 hours (*n* = 3 samples). **g-h**, Flow cytometry dot plots (**g**) and frequencies (**h**, *n* = 6 samples) of IFN-γ production in human CD8 T cells stimulated by plate-coated anti-CD3 (10 μg ml^{-1}) and anti-CD28 (10 μg ml^{-1}) in the presence of DMSO or ZCL278. Data are representative of (**c, e, g**) or pooled from (**b, d, h**) two independent experiments. Data are represented as mean ± SEM, and were analyzed by one-way ANOVA (**a, f**) and two-tailed unpaired Student's t-test (**b, d, h**). Source data are provided as a Source Data file.

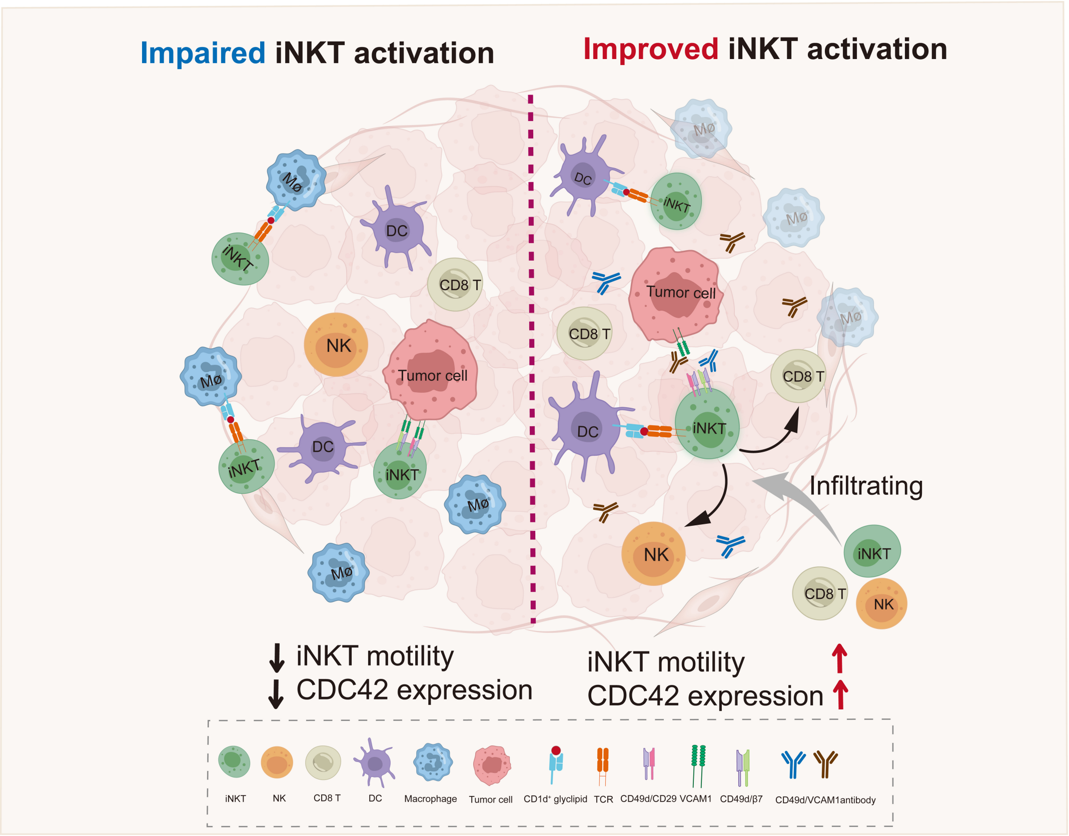

Supplementary Fig. 8 Motility and tumor infiltration are key aspects influencing anti-tumor function of iNKT cells. Macrophage-iNKT cell interactions and VCAM1 signaling inhibit infiltration and activation of iNKT cells in tumor. Interfering with macrophage-iNKT cell interactions and blocking VCAM1 signaling can improve efficacy of iNKT cell-based immunotherapy.

Supplementary Table. Antibodies used for experiments

Antibodies	Clone	Supplier	Cat. No.	Dilution
**Mouse antibodies**				
Purified anti-mouse CD16/32	93	BioLegend	101302	1:500
Ultra-LEAF Purified anti-mouse CD106 (VCAM1)	429	BioLegend	105728	1:100
Ultra-LEAF Purified anti-mouse CD49d	9C10	BioLegend	103709	1:100
Ultra-LEAF Purified Rat IgG2a	RTK2758	BioLegend	400544	1/100
Ultra-LEAF Purified anti-mouse CD3ε	145-2C11	BioLegend	100340	1:100
Ultra-LEAF Purified anti-mouse CD28	37.51	BioLegend	102116	1:100
FITC anti-mouse IA/IE (MHC II)	M5/114.15.2	BioLegend	107606	1:500
FITC anti-mouse CD24	M1/69	BioLegend	101806	1:500
PerCP/Cy5.5 anti-mouse CD45.2	104	BioLegend	109828	1:200
PE anti-mouse CD49d	R1-2	BioLegend	103608	1:200
PE anti-mouse CD11a/CD18 (LFA-1)	H155-78	BioLegend	141006	1:200
PE anti-mouse CD1d	1B1	BioLegend	123510	1:200
PE anti-mouse CD24	M1/69	BioLegend	101808	1:200
PE anti-mouse IA/IE (MHC II)	M5/114.15.2	BioLegend	107608	1:200
PE/Cy7 anti-mouse Ly-6C	HK1.4	BioLegend	128018	1:200
PE/Cy7 anti-mouse IFN-γ	XMG1.2	BioLegend	505826	1:200
PE/Cy7 anti-mouse CD54 (ICAM1)	YN1/1.7.4	BioLegend	116122	1:200
PE/Cy7 anti-mouse F4/80	BM8	BioLegend	123114	1:200
APC anti-mouse CD106 (VCAM1)	429	BioLegend	105718	1:200
APC anti-mouse CD11c	N418	BioLegend	117310	1:200
Alexa Fluor 700 anti-mouse CD4	GK1.5	BioLegend	100430	1:200
APC/Cy7 anti-mouse NK1.1	PK136	BioLegend	108724	1:200
APC/Cy7 anti-mouse CD11c	N418	BioLegend	117324	1:200
APC/Cy7 anti-mouse Ly-6C	HK1.4	BioLegend	128026	1:200
Pacific Blue anti-mouse TCRβ	H57-597	BioLegend	109226	1:500
Pacific Blue anti-mouse F4/80	BM8	BioLegend	123124	1:500
Pacific Blue anti-mouse CD11b	M1/70	BioLegend	101224	1:500
BV510 anti-mouse B220	RA3-6B2	BioLegend	103248	1:200
BV510 anti-mouse CD11b	M1/70	BioLegend	101263	1:200
BV510 anti-mouse IFN-γ	XMG1.2	BioLegend	505842	1:200
BV510 anti-mouse IA/IE (MHC II)	M5/114.15.2	BioLegend	107636	1:200
BV605 anti-mouse CD8α	53-6.7	BioLegend	100743	1:200
BV650 anti-mouse CD206	C068C2	BioLegend	141723	1:200
InVivoMAb anti-mouse CD49d	PS/2	BioXcell	BE0071	10 mg kg-1
InVivoMAb anti-mouse CD106 (VCAM1)	M/K-2.7	BioXcell	BE0027	10 mg kg-1
InVivoMAb rat IgG2b isotype control	LTF-2	BioXcell	BE0090	10 mg kg-1
InVivoMAb rat IgG1 isotype control	HRPN	BioXcell	BE0088	10 mg kg-1

**Human antibodies**				
Purified anti-human CD106 (VCAM1)	STA	BioLegend	305802	1:100
Purified Mouse IgG1	MOPC-21	BioLegend	400101	1:100
Purified anti-human CD3	OKT3	BioLegend	317326	1:100
Purified anti-human CD28	CD28.2	BioLegend	302934	1:100
FITC anti-human CD3	OKT3	BioLegend	317306	1:100
PE anti-human IFN-γ	4S.B3	BioLegend	502509	1:100
PE/Cy7 anti-human CD49d	9F10	BioLegend	304314	1:100
APC anti-human CD106 (VCAM1)	STA	BioLegend	305810	1:100
Alexa Fluor 700 anti-human IFN-γ	4S.B3	BioLegend	502520	1:100
BV510 anti-human CD8	SK1	BioLegend	344732	1:100
**Others**				
PE Donkey anti-rabbit IgG	Poly4064	BioLegend	406421	1:200
anti-CDC42	EPR15620	Abcam	ab187643	1:400
anti-Src (phospho Y419)	EPR17734	Abcam	ab185617	1:400
APC mCD1d-PBS57 tetramer	NIH Tetramer Core Facility			1:2000
APC hCD1d-PBS57 tetramer	NIH Tetramer Core Facility			1:2000
