# Supplementary material for: Motility and tumor infiltration are key aspects of invariant natural killer T cell anti-tumor function
Source: Nat Commun. 2024 Feb 9;15:1213. doi: 10.1038/s41467-024-45208-z (PMC10853287; doi:10.1038/s41467-024-45208-z)
Supplement: Supplementary file 3 — Description of Additional Supplementary Files [file 41467_2024_45208_MOESM3_ESM.pdf]

Description of Additional Supplementary Files

File Name: Supplementary Movie 1
Description: **Host iNKT cell motility at MC38-mCherry tumor periphery and inside tumor.** Two-photon intravital imaging of MC38-mCherry tumor in *Vα14* Tg *Cxcr6^{Gfp}* mouse. MC38 tumor cells expressed mCherry and host iNKT cells expressed GFP. Elapsed time is shown as h:mm:ss.000.

File Name: Supplementary Movie 2
Description: **Transferred iNKT cell motility at MC38-mCherry tumor periphery and inside tumor of 10 days old.** Two-photon intravital imaging of 10 days MC38-mCherry tumor in WT mouse. MC38 tumor cells expressed mCherry and transferred iNKT cells expressed GFP. Elapsed time is shown as h:mm:ss.000.

File Name: Supplementary Movie 3
Description: **Transferred iNKT cell motility at MC38-mCherry tumor periphery and inside tumor of 5 weeks old.** Two-photon intravital imaging of ~5 weeks MC38-mCherry tumor in WT mouse. MC38 tumor cells expressed mCherry and transferred iNKT cells expressed GFP. Elapsed time is shown as h:mm:ss.000.

File Name: Supplementary Movie 4
Description: **Transferred iNKT cell motility at B16F10-mCherry tumor periphery and inside tumor of 10 days old.** Two-photon intravital imaging of 10 days B16F10-mCherry tumor in WT mouse. B16F10 tumor cells expressed mCherry and transferred iNKT cells expressed GFP. Elapsed time is shown as h:mm:ss.000.

File Name: Supplementary Movie 5
Description: **Transferred iNKT cell motility in tumors in *Lyz2^{cre} Cd1d1$^{fl/fl}$* mouse and *Cd1d1$^{fl/fl}$* mouse.** Two-photon intravital imaging of transferred iNKT cells in tumors in *Lyz2^{cre} Cd1d1$^{fl/fl}$* mouse and *Cd1d1$^{fl/fl}$* mouse. MC38 tumor cells expressed mCherry and transferred iNKT cells expressed GFP. Elapsed time is shown as h:mm:ss.000.

File Name: Supplementary Movie 6
Description: **Transferred iNKT cell motility in *Vcam1* knockdown MC38-mCherry tumor and NTC MC38-mCherry tumor.** Two-photon intravital imaging of transferred iNKT cells in *Vcam1* knockdown MC38-mCherry tumor and in NTC MC38-mCherry tumor. MC38 tumor cells expressed mCherry and transferred iNKT cells expressed GFP. Elapsed time is shown as h:mm:ss.000.
