# Supplementary material for: Motility and tumor infiltration are key aspects of invariant natural killer T cell anti-tumor function
Source: Nat Commun. 2024 Feb 9;15:1213. doi: 10.1038/s41467-024-45208-z (PMC10853287; doi:10.1038/s41467-024-45208-z)
Supplement: Supplementary file 10 — Reporting Summary [file 41467_2024_45208_MOESM10_ESM.pdf]

nature portfolio

Corresponding author(s): Li Bai

Last updated by author(s): Dec 15, 2023

Reporting Summary

Nature Portfolio wishes to improve the reproducibility of the work that we publish. This form provides structure for consistency and transparency in reporting. For further information on Nature Portfolio policies, see our Editorial Policies and the Editorial Policy Checklist.

Statistics

For all statistical analyses, confirm that the following items are present in the figure legend, table legend, main text, or Methods section.

n/a	Confirmed	
☐	☒	The exact sample size (*n*) for each experimental group/condition, given as a discrete number and unit of measurement
☐	☒	A statement on whether measurements were taken from distinct samples or whether the same sample was measured repeatedly
☐	☒	The statistical test(s) used AND whether they are one- or two-sided<br>*Only common tests should be described solely by name; describe more complex techniques in the Methods section.*
☒	☐	A description of all covariates tested
☐	☒	A description of any assumptions or corrections, such as tests of normality and adjustment for multiple comparisons
☐	☒	A full description of the statistical parameters including central tendency (e.g. means) or other basic estimates (e.g. regression coefficient) AND variation (e.g. standard deviation) or associated estimates of uncertainty (e.g. confidence intervals)
☐	☒	For null hypothesis testing, the test statistic (e.g. *F*, *t*, *r*) with confidence intervals, effect sizes, degrees of freedom and *P* value noted<br>*Give P values as exact values whenever suitable.*
☒	☐	For Bayesian analysis, information on the choice of priors and Markov chain Monte Carlo settings
☒	☐	For hierarchical and complex designs, identification of the appropriate level for tests and full reporting of outcomes
☐	☒	Estimates of effect sizes (e.g. Cohen's *d*, Pearson's *r*), indicating how they were calculated

Our web collection on statistics for biologists contains articles on many of the points above.

Software and code

Policy information about availability of computer code

Data collection	Tissue images and in vitro cell tracking were taken with Zeiss LSM 980 microscope.<br>The intravital images were taken with Olympus FVMPE-RS two-photon microscope.<br>Flow cytometry data were acquired with BD FACSVerse flow cytometer or BECKMAN CytoFLEX S flow cytometer.<br>qRT-PCR data were taken with Roche LightCycler 96.
Data analysis	Images were analyzed with ImageJ software (Vesion 2.1.0/1.53c, National Institutes of Health).<br>Flow cytometry data were analyzed with FlowJo software (Version 10.5.0, Tree Star, Inc., Ashland, OR).<br>Intravital imaging data were analyzed with Imaris software (Version 9.2.0, Bitplane).<br>Prism 8 (Version 8.3.1, GraphPad) was used to perform statistical analyses.

For manuscripts utilizing custom algorithms or software that are central to the research but not yet described in published literature, software must be made available to editors and reviewers. We strongly encourage code deposition in a community repository (e.g. GitHub). See the Nature Portfolio guidelines for submitting code & software for further information.

Data

Policy information about availability of data

All manuscripts must include a data availability statement. This statement should provide the following information, where applicable:

- Accession codes, unique identifiers, or web links for publicly available datasets
- A description of any restrictions on data availability
- For clinical datasets or third party data, please ensure that the statement adheres to our policy

RNA-seq datasets reported in this study have been deposited in the National Center for Biotechnology Information Sequence Read Archive under accession number PRJNA946041. Other data supporting our findings are available in the Source data file. Source data are provided with this paper.

Research involving human participants, their data, or biological material

Policy information about studies with human participants or human data. See also policy information about sex, gender (identity/presentation), and sexual orientation and race, ethnicity and racism.

Reporting on sex and gender	We obtained PBMCs from blood of healthy volunteers irrespective of sex and gender.
Reporting on race, ethnicity, or other socially relevant groupings	We obtained PBMCs from blood of healthy volunteers irrespective of race, thnicity, or other socially relrvant groupings.
Population characteristics	The volunteers were healthy irrespective of age.
Recruitment	The healthy volunteers were recruitments randomly, and no self-selection bias affected the recruitment.
Ethics oversight	PBMCs from blood of healthy volunteers were obtained in accordance with protocols approved by the Biomedical Ethics Committee of the Institute of Health and Medicine of Hefei comprehensive national Science Center (YXLL-2023001).

Note that full information on the approval of the study protocol must also be provided in the manuscript.

Field-specific reporting

Please select the one below that is the best fit for your research. If you are not sure, read the appropriate sections before making your selection.

☒ Life sciences ☐ Behavioural & social sciences ☐ Ecological, evolutionary & environmental sciences

For a reference copy of the document with all sections, see nature.com/documents/nr-reporting-summary-flat.pdf

Life sciences study design

All studies must disclose on these points even when the disclosure is negative.

Sample size	No prior sample size calculation was performed, and sample sizes in the experiments were set based on our previous experience and similar studies in the literature.
Data exclusions	No data were excluded from the analysis.
Replication	All experiments were repeated at least twice, and each result are similar.
Randomization	All mice, cell lines and PBMC from healthy volunteers were grouped randomly.
Blinding	The investigators were blinded to group allocation during data collection and analysis.

Reporting for specific materials, systems and methods

We require information from authors about some types of materials, experimental systems and methods used in many studies. Here, indicate whether each material, system or method listed is relevant to your study. If you are not sure if a list item applies to your research, read the appropriate section before selecting a response.

Materials & experimental systems

n/a	Involved in the study	
☐	☒	Antibodies
☐	☒	Eukaryotic cell lines
☒	☐	Palaeontology and archaeology
☐	☒	Animals and other organisms
☒	☐	Clinical data
☒	☐	Dual use research of concern
☒	☐	Plants

Methods

n/a	Involved in the study	
☒	☐	ChIP-seq
☐	☒	Flow cytometry
☒	☐	MRI-based neuroimaging

Antibodies

Antibodies used

Mouse antibodies
Purified anti-mouse CD16/32 93 BioLegend 101302 1:500
Ultra-LEAF Purified anti-mouse CD106 429 BioLegend 105728 1:100
Ultra-LEAF Purified anti-mouse CD49d 9C10 BioLegend 103709 1:100
Ultra-LEAF Purified Rat IgG2a RTK2758 BioLegend 400544 1:100
Ultra-LEAF Purified anti-mouse CD3e 145-2C11 BioLegend 100340 1:100
Ultra-LEAF Purified anti-mouse CD28 37.51 BioLegend 102116 1:100
FITC anti-mouse IA/IE M5/114.15.2 BioLegend 107606 1:500
FITC anti-mouse CD24 M1/69 BioLegend 101806 1:500
PerCP/Cy5.5 anti-mouse CD45.2 104 BioLegend 109828 1:200
PE anti-mouse CD49d R1-2 BioLegend 103608 1:200
PE anti-mouse CD11a/CD18 H155-78 BioLegend 141006 1:200
PE anti-mouse CD1d 1B1 BioLegend 123510 1:200
PE anti-mouse CD24 M1/69 BioLegend 101808 1:200
PE anti-mouse IA/IE M5/114.15.2 Biolegend 107608 1:200
PE/Cy7 anti-mouse Ly-6C HK1.4 BioLegend 128018 1:200
PE/Cy7 anti-mouse IFN-g XMG1.2 BioLegend 505826 1:200
PE/Cy7 anti-mouse CD54 YN1/1.7.4 BioLegend 116122 1:200
PE/Cy7 anti-mouse F4/80 BM8 BioLegend 123114 1:200
APC anti-mouse CD106 429 BioLegend 105718 1:200
APC anti-mouse CD11c N418 BioLegend 117310 1:200
Alexa Fluor 700 anti-mouse CD4 GK1.5 Biolegend 100430 1:200
APC/Cy7 anti-mouse NK1.1 PK136 BioLegend 108724 1:200
APC/Cy7 anti-mouse CD11c N418 BioLegend 117324 1:200
APC/Cy7 anti-mouse Ly-6C HK1.4 BioLegend 128026 1:200
Pacific Blue anti-mouse TCRb H57-597 BioLegend 109226 1:500
Pacific Blue anti-mouse F4/80 BM8 BioLegend 123124 1:500
Pacific Blue anti-mouse CD11b M1/70 BioLegend 101224 1:500
BV510 anti-mouse B220 RA3-6B2 BioLegend 103248 1:200
BV510 anti-mouse CD11b M1/70 BioLegend 101263 1:200
BV510 anti-mouse IFN-g XMG1.2 BioLegend 505842 1:200
BV510 anti-mouse IA/IE M5/114.15.2 Biolegend 107636 1:200
BV605 anti-mouse CD8a 53-6.7 BioLegend 100743 1:200
BV650 anti-mouse CD206 C068C2 Biolegend 141723 1:100
InVivoMAb anti-mouse CD49d PS/2 BioXCell BE0071 (10 mg kg-1)
InVivoMAb anti-mouse CD106 M/K-2.7 BioXCell BE0027 (10 mg kg-1)
InVivoMAb rat IgG2b isotype control LTF-2 BioXCell BE0090 (10 mg kg-1)
InVivoMAb rat IgG1 isotype control HRPN BioXCell BE0088 (10 mg kg-1)
Human antibodies
Purified anti-human CD106 STA BioLegend 305802 1:100
Purified Mouse IgG1 MOPC-21 BioLegend 400101 1:100
Purified anti-human CD3 OKT3 BioLegend 317326 1:100
Purified anti-human CD28 CD28.2 BioLegend 302934 1:100
FITC anti-human CD3 OKT3 BioLegend 317306 1:100
PE anti-human IFNg 4S.B3 BioLegend 502509 1:100
PE/Cy7 anti-human CD49d 9F10 BioLegend 304314 1:100
APC anti-human CD106 STA BioLegend 305810 1:100
Alexa Fluor 700 anti-human IFN-g 4S.B3 BioLegend 502520 1:100
BV510 anti-human CD8 SK1 BioLegend 344732 1:100
Others
PE Donkey anti-rabbit IgG Poly4064 BioLegend 406421 1:200
anti-CDC42 EPR15620 Abcam ab187643 1:400
anti-Src (phospho Y419) EPR17734 Abcam 185617 1:400
APC mCD1d-PBS57 tetramer NIH Tetramer Core Facility 1:2000
APC hCD1d-PBS57 tetramer NIH Tetramer Core Facility 1:2000

Validation

All antibodies used in the study are commercially available and validated. The validation data for staining of cells performed by manufacturers can be acquired at their website. The concentrations of antibodies used in the study were confirmed on the basis of

recommended concentrations and concentration test experiments.
Mouse
Purified anti-mouse CD16/32 (https://www.biolegend.com/en-us/products/purified-anti-mouse-cd16-32-antibody-190?pdf=true&displayInline=true&leftRightMargin=15&topBottomMargin=15&filename=Purified%20anti-mouse%20CD16/32%20Antibody.pdf&v=20230726063409)
Ultra-LEAF Purified anti-mouse CD106 (https://www.biolegend.com/en-us/products/ultra-leaf-purified-anti-mouse-cd106-antibody-18659?pdf=true&displayInline=true&leftRightMargin=15&topBottomMargin=15&filename=Ultra-LEAF™%20Purified%20anti-mouse%20CD106%20Antibody.pdf&v=20230816063029)
Ultra-LEAF Purified anti-mouse CD49d (https://www.biolegend.com/en-us/products/ultra-leaf-purified-anti-mouse-cd49d-antibody-18786?pdf=true&displayInline=true&leftRightMargin=15&topBottomMargin=15&filename=Ultra-LEAF™%20Purified%20anti-mouse%20CD49d%20Antibody.pdf&v=20230816063029)
Ultra-LEAF Purified Rat IgG2a (https://www.biolegend.com/en-us/products/ultra-leaf-purified-rat-igg2a-kappa-isotype-ctrl-7726)
Ultra-LEAF Purified anti-mouse CD3e (https://www.biolegend.com/en-us/products/ultra-leaf-purified-anti-mouse-cd3epsilon-antibody-7722?pdf=true&displayInline=true&leftRightMargin=15&topBottomMargin=15&filename=Ultra-LEAF™%20Purified%20anti-mouse%20CD3ε%20Antibody.pdf&v=20231207073024)
Ultra-LEAF Purified anti-mouse CD28 (https://www.biolegend.com/en-us/products/ultra-leaf-purified-anti-mouse-cd28-antibody-7733?pdf=true&displayInline=true&leftRightMargin=15&topBottomMargin=15&filename=Ultra-LEAF™%20Purified%20anti-mouse%20CD28%20Antibody.pdf&v=20231207073024)
FITC anti-mouse IA/IE (https://www.biolegend.com/en-us/products/fitc-anti-mouse-i-a-i-e-antibody-366?pdf=true&displayInline=true&leftRightMargin=15&topBottomMargin=15&filename=FITC%20anti-mouse%20I-A/I-E%20Antibody.pdf&v=20230714033116)
FITC anti-mouse CD24 (https://www.biolegend.com/en-us/products/fitc-anti-mouse-cd24-antibody-341?pdf=true&displayInline=true&leftRightMargin=15&topBottomMargin=15&filename=FITC%20anti-mouse%20CD24%20Antibody.pdf&v=20230726063409)
PerCP/Cy5.5 anti-mouse CD45.2 (https://www.biolegend.com/en-us/products/percp-cyanine5-5-anti-mouse-cd452-antibody-4271?pdf=true&displayInline=true&leftRightMargin=15&topBottomMargin=15&filename=PerCP/Cyanine5.5%20anti-mouse%20CD45.2%20Antibody.pdf&v=20231114073227)
PE anti-mouse CD49d (https://www.biolegend.com/en-us/products/pe-anti-mouse-cd49d-antibody-440?pdf=true&displayInline=true&leftRightMargin=15&topBottomMargin=15&filename=PE%20anti-mouse%20CD49d%20Antibody.pdf&v=20230726063409)
PE anti-mouse CD11a/CD18 (https://www.biolegend.com/en-us/products/pe-anti-mouse-cd11a-cd18-lfa-1-antibody-7042?pdf=true&displayInline=true&leftRightMargin=15&topBottomMargin=15&filename=PE%20anti-mouse%20CD11a/CD18%20(LFA-1)%20Antibody.pdf&v=20231114073227)
PE anti-mouse CD1d (https://www.biolegend.com/en-us/products/pe-anti-mouse-cd1d-cd1-1-ly-38-antibody-4320?pdf=true&displayInline=true&leftRightMargin=15&topBottomMargin=15&filename=PE%20anti-mouse%20CD1d%20(CD1.1,%20Ly-38)%20Antibody.pdf&v=20230817033058)
PE anti-mouse CD24 (https://www.biolegend.com/en-us/products/pe-anti-mouse-cd24-antibody-343?pdf=true&displayInline=true&leftRightMargin=15&topBottomMargin=15&filename=PE%20anti-mouse%20CD24%20Antibody.pdf&v=20230726063409)
PE anti-mouse IA/IE (https://www.biolegend.com/en-us/products/pe-anti-mouse-i-a-i-e-antibody-367?pdf=true&displayInline=true&leftRightMargin=15&topBottomMargin=15&filename=PE%20anti-mouse%20I-A/I-E%20Antibody.pdf&v=20231114073227)
PE/Cy7 anti-mouse Ly-6C (https://www.biolegend.com/en-us/products/pe-cyanine7-anti-mouse-ly-6c-antibody-6063?pdf=true&displayInline=true&leftRightMargin=15&topBottomMargin=15&filename=PE/Cyanine7%20anti-mouse%20Ly-6C%20Antibody.pdf&v=20230920123134)
PE/Cy7 anti-mouse IFN-g (https://www.biolegend.com/en-us/products/pe-cyanine7-anti-mouse-ifn-gamma-antibody-5865?pdf=true&displayInline=true&leftRightMargin=15&topBottomMargin=15&filename=PE/Cyanine7%20anti-mouse%20IFN-γ%20Antibody.pdf&v=20230726063409)
PE/Cy7 anti-mouse CD54 (https://www.biolegend.com/en-us/products/pe-cyanine7-anti-mouse-cd54-antibody-14759?pdf=true&displayInline=true&leftRightMargin=15&topBottomMargin=15&filename=PE/Cyanine7%20anti-mouse%20CD54%20Antibody.pdf&v=20230726063409)
PE/Cy7 anti-mouse F4/80 (https://www.biolegend.com/en-us/products/pe-cyanine7-anti-mouse-f4-80-antibody-4070?pdf=true&displayInline=true&leftRightMargin=15&topBottomMargin=15&filename=PE/Cyanine7%20anti-mouse%20F4/80%20Antibody.pdf&v=20230114013553)
APC anti-mouse CD106 (https://www.biolegend.com/en-us/products/apc-anti-mouse-cd106-antibody-6079?pdf=true&displayInline=true&leftRightMargin=15&topBottomMargin=15&filename=APC%20anti-mouse%20CD106%20Antibody.pdf&v=20230920123134)
APC anti-mouse CD11c (https://www.biolegend.com/en-us/products/apc-anti-mouse-cd11c-antibody-1813?pdf=true&displayInline=true&leftRightMargin=15&topBottomMargin=15&filename=APC%20anti-mouse%20CD11c%20Antibody.pdf&v=20230114013553)
Alexa Fluor 700 anti-mouse CD4 (https://www.biolegend.com/en-us/products/alexa-fluor-700-anti-mouse-cd4-antibody-3385?pdf=true&displayInline=true&leftRightMargin=15&topBottomMargin=15&filename=Alexa%20Fluor®%20700%20anti-mouse%20CD4%20Antibody.pdf&v=20230726063409)
APC/Cy7 anti-mouse NK1.1 (https://www.biolegend.com/en-us/products/apc-cyanine7-anti-mouse-nk-1-1-antibody-4002?pdf=true&displayInline=true&leftRightMargin=15&topBottomMargin=15&filename=APC/Cyanine7%20anti-mouse%20NK-1.1%20Antibody.pdf&v=20230726063409)
APC/Cy7 anti-mouse CD11c (https://www.biolegend.com/en-us/products/apc-cyanine7-anti-mouse-cd11c-antibody-3931?pdf=true&displayInline=true&leftRightMargin=15&topBottomMargin=15&filename=APC/Cyanine7%20anti-mouse%20CD11c%20Antibody.pdf&v=20230114013553)
APC/Cy7 anti-mouse Ly-6C (https://www.biolegend.com/en-us/products/apc-cyanine7-anti-mouse-ly-6c-antibody-6758?pdf=true&displayInline=true&leftRightMargin=15&topBottomMargin=15&filename=APC/Cyanine7%20anti-mouse%20Ly-6C%20Antibody.pdf&v=20230920123134)
Pacific Blue anti-mouse TCRb (https://www.biolegend.com/en-us/products/pacific-blue-anti-mouse-tcr-beta-chain-antibody-4538?pdf=true&displayInline=true&leftRightMargin=15&topBottomMargin=15&filename=Pacific%20Blue™%20anti-mouse%20TCR%20β%20chain%20Antibody.pdf&v=20230801063041)
Pacific Blue anti-mouse F4/80 (https://www.biolegend.com/en-us/products/pacific-blue-anti-mouse-f4-80-antibody-4075?

pdf=true&displayInline=true&leftRightMargin=15&topBottomMargin=15&filename=Pacific%20Blue™%20anti-mouse%20F4/80%20Antibody.pdf&v=20230114013553)
Pacific Blue anti-mouse CD11b (https://www.biolegend.com/en-us/products/pacific-blue-anti-mouse-human-cd11b-antibody-3863?pdf=true&displayInline=true&leftRightMargin=15&topBottomMargin=15&filename=Pacific%20Blue™%20anti-mouse/human%20CD11b%20Antibody.pdf&v=20231114073227)
BV510 anti-mouse B220 (https://www.biolegend.com/en-us/products/brilliant-violet-510-anti-mouse-human-cd45r-b220-antibody-7996?pdf=true&displayInline=true&leftRightMargin=15&topBottomMargin=15&filename=Brilliant%20Violet%20510™%20anti-mouse/human%20CD45R/B220%20Antibody.pdf&v=20230803063053)
BV510 anti-mouse CD11b (https://www.biolegend.com/en-us/products/brilliant-violet-510-anti-mouse-human-cd11b-antibody-7993?pdf=true&displayInline=true&leftRightMargin=15&topBottomMargin=15&filename=Brilliant%20Violet%20510™%20anti-mouse/human%20CD11b%20Antibody.pdf&v=20230726063409)
BV510 anti-mouse IFN-g (https://www.biolegend.com/en-us/products/brilliant-violet-510-anti-mouse-ifn-gamma-antibody-8610?pdf=true&displayInline=true&leftRightMargin=15&topBottomMargin=15&filename=Brilliant%20Violet%20510™%20anti-mouse%20IFN-γ%20Antibody.pdf&v=20230726063409)
BV510 anti-mouse IA/IE (https://www.biolegend.com/en-us/products/brilliant-violet-510-anti-mouse-i-a-i-e-antibody-7997?pdf=true&displayInline=true&leftRightMargin=15&topBottomMargin=15&filename=Brilliant%20Violet%20510™%20anti-mouse%20I-A/I-E%20Antibody.pdf&v=20230817033058)
BV605 anti-mouse CD8a (https://www.biolegend.com/en-us/products/brilliant-violet-605-anti-mouse-cd8a-antibody-7636?pdf=true&displayInline=true&leftRightMargin=15&topBottomMargin=15&filename=Brilliant%20Violet%20605™%20anti-mouse%20CD8a%20Antibody.pdf&v=20230714033116)
BV650 anti-mouse CD206 (https://www.biolegend.com/en-us/products/brilliant-violet-650-anti-mouse-cd206-mmr-antibody-8842?pdf=true&displayInline=true&leftRightMargin=15&topBottomMargin=15&filename=Brilliant%20Violet%20650™%20anti-mouse%20CD206%20(MMR)%20Antibody.pdf&v=20230817033058)
InVivoMAb anti-mouse CD49d (https://bioxcell.com/invivomab-anti-mouse-human-vla-4-cd49d-be0071)
InVivoMAb anti-mouse CD106 (https://bioxcell.com/invivomab-anti-mouse-cd106-vcam-1-be0027)
InVivoMAb rat IgG2b isotype control LTF-2 (https://bioxcell.com/invivomab-rat-igg2b-isotype-control-anti-keyhole-limpet-hemocyanin-be0090)
InVivoMAb rat IgG1 isotype control HRPN (https://bioxcell.com/invivomab-rat-igg1-isotype-control-anti-horseradish-peroxidase-be0088)
Human antibodies
Purified anti-human CD106 (https://www.biolegend.com/en-us/products/purified-anti-human-cd106-antibody-844?pdf=true&displayInline=true&leftRightMargin=15&topBottomMargin=15&filename=Purified%20anti-human%20CD106%20Antibody.pdf&v=20230726063409)
Purified Mouse IgG1 (https://www.biolegend.com/en-us/products/purified-mouse-igg1-kappa-isotype-ctrl-1375?pdf=true&displayInline=true&leftRightMargin=15&topBottomMargin=15&filename=Purified%20Mouse%20IgG1,%20κ%20Isotype%20Ctrl%20Antibody.pdf&v=20230714033116)
Purified anti-human CD3 (https://www.biolegend.com/en-us/products/ultra-leaf-purified-anti-human-cd3-antibody-7745?pdf=true&displayInline=true&leftRightMargin=15&topBottomMargin=15&filename=Ultra-LEAF™%20Purified%20anti-human%20CD3%20Antibody.pdf&v=20231207073024)
Purified anti-human CD28 (https://www.biolegend.com/en-us/products/ultra-leaf-purified-anti-human-cd28-antibody-7743?pdf=true&displayInline=true&leftRightMargin=15&topBottomMargin=15&filename=Ultra-LEAF™%20Purified%20anti-human%20CD28%20Antibody.pdf&v=20231207073024)
FITC anti-human CD3 OKT3 (https://www.biolegend.com/en-us/products/fitc-anti-human-cd3-antibody-3644?pdf=true&displayInline=true&leftRightMargin=15&topBottomMargin=15&filename=FITC%20anti-human%20CD3%20Antibody.pdf&v=20230726063409)
PE anti-human IFNg (https://www.biolegend.com/en-us/products/fitc-anti-human-cd3-antibody-3644?pdf=true&displayInline=true&leftRightMargin=15&topBottomMargin=15&filename=FITC%20anti-human%20CD3%20Antibody.pdf&v=20230726063409)
PE/Cy7 anti-human CD49d (https://www.biolegend.com/en-us/products/pe-cyanine7-anti-human-cd49d-antibody-6776?pdf=true&displayInline=true&leftRightMargin=15&topBottomMargin=15&filename=PE/Cyanine7%20anti-human%20CD49d%20Antibody.pdf&v=20231114073227)
APC anti-human CD106 (https://www.biolegend.com/en-us/products/apc-anti-human-cd106-antibody-840?pdf=true&displayInline=true&leftRightMargin=15&topBottomMargin=15&filename=APC%20anti-human%20CD106%20Antibody.pdf&v=20231114073227)
Alexa Fluor 700 anti-human IFN-g (https://www.biolegend.com/en-us/products/alexa-fluor-700-anti-human-ifn-gamma-antibody-3440?pdf=true&displayInline=true&leftRightMargin=15&topBottomMargin=15&filename=Alexa%20Fluor®%20700%20anti-human%20IFN-γ%20Antibody.pdf&v=20230628033023)
BV510 anti-human CD8 (https://www.biolegend.com/en-us/products/brilliant-violet-510-anti-human-cd8-antibody-10739?pdf=true&displayInline=true&leftRightMargin=15&topBottomMargin=15&filename=Brilliant%20Violet%20510™%20anti-human%20CD8%20Antibody.pdf&v=20231206085752)
Others
anti-CDC42 EPR15620 (https://www.abcam.cn/products/primary-antibodies/cdc42-antibody-epr15620-ab187643.html)
anti-Src (phospho Y419) EPR17734 (https://www.abcam.cn/products/primary-antibodies/src-phospho-y419-antibody-epr17734-ab185617.html)

Eukaryotic cell lines

Policy information about cell lines and Sex and Gender in Research

Cell line source(s)
MC38, B16F10, MDA-MB-231, HeLa were provided by cooperator. We overexpressed mCherry and CD1d in MC38, hVCAM1 and hCD1d in MDA-MB-231, hCD1d in HeLa. We knocked down Vcam1 in MC38, MC38-mCherry, MC38.CD1d.

Authentication
We used morphology analysis with microscopy to authenticate cell lines.

Mycoplasma contamination	We had not tested mycoplasma contamination.
Commonly misidentified lines (See ICLAC register)	We had not used any commonly misidentified lines.

Animals and other research organisms

Policy information about studies involving animals; ARRIVE guidelines recommended for reporting animal research, and Sex and Gender in Research

Laboratory animals	C57BL/6 mice were purchased from the Gempharmatech Co., Ltd. Cd11ccre mice, Lyz2cre mice, Va14 Tg Cxcr6Gfp mice, and Cd1d1fl/fl mice have previously been described41 and were provided by Dr Albert Bendelac. Ai9 mice were provided by Dr Tian Xue in University of Science and Technology of China. All mice were on the C57BL/6 background. In our study, we used littermate controls and sex-matched mice at 6-12 weeks old.
Wild animals	The study did not involve wild animals.
Reporting on sex	Both male and female mice were used.
Field-collected samples	The study did not involve samples collected from the field.
Ethics oversight	All animal procedures were approved by the Institutional Animal Care and Use Committee of University of Science and Technology of China, and experiments were performed in accordance with the approved guidelines (USTCACUC23120122033).

Note that full information on the approval of the study protocol must also be provided in the manuscript.

Flow Cytometry

Plots

Confirm that:

☒ The axis labels state the marker and fluorochrome used (e.g. CD4-FITC).

☒ The axis scales are clearly visible. Include numbers along axes only for bottom left plot of group (a 'group' is an analysis of identical markers).

☒ All plots are contour plots with outliers or pseudocolor plots.

☒ A numerical value for number of cells or percentage (with statistics) is provided.

Methodology

Sample preparation	Tissues were collected from tumor-bearing mice or WT mice. Splenic lymphocytes were collected by erythrocyte lysis. Hepatic lymphoctes were collected by Percoll density gradient centrifugation. After digestion of tumor tissue, intratumoral lymphocytes were collected by Percoll density gradient centrifugation.
Instrument	Flow cytometry data were acquired with BD FACSVerse flow cytometer or BECKMAN CytoFLEX S flow cytometer.
Software	Flow cytometry data were analyzed with FlowJo software (Version 10.5.0, Tree Star, Inc., Ashland, OR).
Cell population abundance	In RNA-seq experiment and living imaging of human iNKT cells, purity of cells after sorting checked by flow cytometry, which purity are over 90%.
Gating strategy	Gating strategy is shown in the Supplementary Figure 1.

☒ Tick this box to confirm that a figure exemplifying the gating strategy is provided in the Supplementary Information.
